# Assistive technologies in healthcare: utilization and healthcare workers perceptions in Germany

**DOI:** 10.1186/s12913-024-12162-x

**Published:** 2025-02-10

**Authors:** Domenic Sommer, Eva Lermer, Florian Wahl, Luis I. Lopera G.

**Affiliations:** 1 Deggendorf Institute of Technology, Dieter-Görlitz-Platz 1, Deggendorf, 94469 Germany; 2 Center for Leadership and People Management, Department Psychology, LMU Munich, Geschwister-Scholl-Platz 1, Munich, 80539 Germany; 3 Department of Business Psychology, Technical University of Applied Sciences Augsburg, An der Hochschule 1, Augsburg, 86161 Germany; 4 Friedrich Alexander University Erlangen-Nuremberg, Henkestrasse 91, Erlangen, 91052 Germany

**Keywords:** Assistive technologies, Healthcare workers, Human-computer interaction, Information and communication technologies, Artificial intelligence

## Abstract

**Background:**

According to the WHO, assistive technology (AT) is defined as the superset of technologies that improve or maintain the functioning of different senses, mobility, self-care, well-being, and inclusion of patients. ATs also include technologies for healthcare workers (HCWs) to reduce workloads and improve efficiency and patient care outcomes. Software ATs for HCWs include communication software, artificial intelligence (AI), text editors, planning tools, decision support systems, and health records. Hardware ATs for HCWs can range from communication devices, sensors, and specialized medical equipment to robots.

**Aims:**

With this indicative study, we explore HCW utilization, perceptions, and adoption barriers of ATs. We emphasize ATs role in enhancing HCWs’ efficiency and effectiveness in healthcare delivery.

**Methods:**

A cross-sectional online survey was conducted through August 2024 with HCWs in Bavaria via a network recruiting approach. We used convenience sampling but ensured that only HCWs were part of our study population. Our survey included (i) usage, (ii) usefulness, and (iii) perceptions regarding ATs. The survey comprised 11 close-ended and three open-ended questions, including story stems evaluated by a deductive qualitative template analysis. Our mixed-method evaluation also employed descriptive and bivariate statistics.

**Results:**

Three hundred seventy-one HCWs (♂63.9 %, ♀36.1 %) participated in our survey, primarily 133 administrators, 116 nurses, and 34 doctors. More than half of the study participants (58.6 %) reported having advanced technical skills. Regarding usage, communication platforms (82.2 %) and communication devices (86 %) were the most commonly used ATs. Advanced ATs such as body-worn sensors, medical devices with interfaces, identification devices, and robots were underutilized in our sample. ATs were reported to be helpful in all job roles but need improvements in capacity and integration. Key barriers to adoption included outdated infrastructure, interoperability, and a lack of training.

**Conclusion:**

Our study suggests that HCWs may want to incorporate ATs into their workflows as they see how, in theory, these technologies would improve HCW’s efficiency, resulting in better patient care. However, to realize this potential, efforts in ATs integration and accessibility are essential. Given this study’s modest sample size and generalizability limitations, further research is needed to explore the adoption, implementation, and impact of ATs in healthcare.

**Supplementary Information:**

The online version contains supplementary material available at 10.1186/s12913-024-12162-x.

## Introduction

This study investigates assistive technologiess (ATs) utilization and perception by healthcare workers (HCWs). Initially, we introduce the topic by defining ATs to facilitate shared understanding. Second, demographic and systemic challenges of HCWs were explored to visualize the relevance of analyzing ATs in healthcare. The solutions for the mentioned challenges are addressed in the third paragraph, which discusses the benefits of ATs. Finally, the scope and lead question are defined to show the focus of this article. Following the introduction, the article adopts a classical structure, encompassing the Methods, Results, Results, and Conclusion sections.

### Ontology of AT in Healthcare

All technologies that support HCWs and their patients, such as monitoring, automation, or decision support, are summarized in this study under the umbrella term AT. AT definitions are primarily focused on aiding vulnerable patients [1, 2]. AT encompasses hardware and software solutions, including robotics and AI-driven systems, designed to assist humans in overcoming impairments like Parkinson ’s [3]. The WHO defines AT as a superset term of technologies that assists patients’ senses, expression, mobility, and decision-making in all areas of life [4]. The International Organization for Standardization (ISO) sees AT as products that improve the functions of persons, reducing impairments and burdens [5]. AT aligns are facilitating the achievement of goals of the UN Convention on the Rights of Persons with Disabilities [6]. Traditionally, the understanding of AT is focused on patients and facilitating and maintaining their functional abilities, senses, and well-being. The mainly patient-centered AT term includes the safe and effective use of tech across various contexts, particularly for enhancing independence and participation [4].

This study broadens the scope of AT to include tech that assist HCWs, to enhance efficiency, autonomy, and ability to deliver high-quality care. In this context, AT is a set of tech designed to achieve beneficial outcomes for both HCWs and patients. Alternative terms to ATs, e.g., digital health technologies, information and communication tech (ICT), or tech-enabled care, weren’t chosen because they are subsumed under AT [7–9]. For HCWs, the goals of AT are to promote independence, safety, communication, inclusion, and healthcare access. ATs, such as communication tools, artificial intelligence (AI), text editors, planning applications, decision support systems, electronic health records, sensors, medical equipment, and robots, are employed by HCWs. While these technologies directly aid HCWs, their use also indirectly improves patient outcomes by streamlining workflows and reducing workload burdens [6].

### Relevance of AT in healthcare

Western societies face HCWs shortages and higher care needs related to demographics, underscoring the need to support HCWs. Germany’s healthcare system faces a peak of elderly needing care in 2040 and fewer young employable HCWs [10]. In addition, economic concerns rise, as Germany in 2022 spent up to 12,7 % of the GDP on healthcare, after the US with the second highest healthcare expenses in OECD [11].

Technology is increasingly important in daily life and offers solutions for the HCWs and their patients who adopt wearables, apps, or voice assistants to manage health conditions [12, 13]. Since the onset of the Coronavirus Disease (COVID-19), HCWs have witnessed an increase in the adoption of ATs [14], underscoring the importance of this study. Crises like the COVID-19 pandemic have profoundly transformed healthcare; for instance, 30.1 % of all outpatient visits in the US during the pandemic were conducted via telemedicine (TMed) [15, 16]. Advances in digital health, such as electronic health records (EHR) and TMed, transformed healthcare delivery. For some areas, like EHR, providers are penalized for not using AT, as funding sees opportunities for patients and cost savings [17]. Wearables and intelligent buildings, such as fall detection systems and motion sensors, become common in nursing homes, enhancing the safety and comfort of patients [18]. Furthermore, artificial intelligence (AI) is increasingly used in nursing and is seen by two-thirds as an opportunity [19]. ATs are not only designed to ensure safety, higher efficiency, easing administration but also to reduce hospital admissions, facilitate health-related communication, and save time on supportive nursing tasks, allowing HCW to focus on complex direct patient care [20–23]. ATs, such as TMeds, improve healthcare access [16, 24]. In many cases, the chances of using ATs in healthcare are enormous, including enhancing patient outcomes, care provision, workloads, and economic costs [25]. In the year 2022, globally, 2.5 billion people require one or more ATs, which shows the impact AT have in society [4].

### Benefits of AT in Healthcare

As depicted in Table 1, various ATs indicate improved outcomes, such as patient portals, TMeds, EHRs, remote monitoring, and sensors [26]. Although this publication focuses on HCWs, the ATs benefits primarily affect patients and HCWs alike.

Studies suggest that ATs, such as patient portals, telemedicine, and EHR, may increase treatment availability and accessibility, particularly in remote or underserved areas [24]. ATs can enhance HCWs internal and intersectoral coordination, collaboration and commmunication [36]. Applying technologies in healthcare can lead to faster and more accurate information flows [9]. ATs such as decision support systems can reduce errors and increase efficiency, including disease self-management [22, 45]. Other ATs such as sensors and automated systems are related to increased safety due to the possibilities of observing health and identifying emergencies like heart attacks or falls [18, 44]. More advanced AT such as AI-driven patient monitoring and route planning spread and HCW recognize opportunities [19]. Patients also anticipate more changes than challenges and expect AT health improvements and well-being [46]. AT can empower patients autonomy and security [8].

### Research Gap and Challenges of AT in Healthcare

Studies regarding the usage of ATs by HCWs remain limited, and the WHO voiced in their first global AT report the need for more research [4]. In Germany, ATs adoption by HCWs has not been thoroughly evaluated in digital transformation [47]. Krick et al. [33] state that studies focusing on evaluation technologies in formal care at a high evidence level are rare. Previous research on the acceptance of certain ATs primarily focuses on the patient perspective [48], while studies on HCWs perceptions mainly concentrate on specific technologies, such as assistive robots, which are emerging as a new development [3]. Studies like Fotteler et al. show the perspective of older adults, who could gain benefits from ATs, such as therapy monitoring, but leave open the implications for HCWs [48]. TMed e.g., has become a well-accepted post-pandemic AT in Germany, although its adoption still requires comprehensive finalization and adequate provider training, which remain incomplete [35]. Challenges, including acceptance, ethics, organization, change management, and training, need to be overcome for AT adaption in healthcare [3, 46, 49]. Integrating both HCW and patients’ perspectives as early as possible is vital in tech implementation, giving stakeholders information, training, and reducing fears [35, 50, 51]. Nevertheless, the practice seems to struggle with the ATs implementation, leading to lost potential due to ATs underutilization [33].

Research regarding the multidimensional usage of AT is necessary to overcome the mentioned challenges. Human-Computer Interaction Research primarily examines specific ATs like decision support systems (DSS), highlighting cost saving, social-economic outcomes, health improvements, and workforce benefits [7, 37, 40, 52]. Most literature focuses on e-health and TMed, even if more solutions are available [47]. Research on the effectiveness of new ATs like AI furthermore is limited, although popularity and end-consumer products [7]. The mixed results regarding the perception of different ATs limit the current state of research. Sommer et al. [19, 43] showed that HCWs well receive ATs in the form of AI and (non-) humanoid service robots. Research shows that AT has the potential to reduce HCW workloads [27]. Other studies see a complicated relationship between HCW and AT because of fears of competition with AT and a potential job replacement by tools such as robots [53]. In addition, Mace et al. [12] state that ageism is a severe problem in AT, leading to attributions that older HCW and seniors cant use ATs even if the user age continuously increases [12].

### Research Aim and Lead Questions

As current research reveals limitations regarding HCW using AT, we aim to explore AT’s potential. We examine the current integration of AT into daily workflows to provide an overview of the current use cases and deliver insights for researchers, HCW, managers, policymakers, and AT developers. We aim to identify the impact, challenges, and potential of ATs as perceived by HCW for Bavaria (Germany). The future role of ATs is part of our study. Our investigated research questions (RQs), as follows, are:

1. To what extent and how are ATs utilized by HCW daily, weekly and monthly?
2. How do HCWs evaluate the impact of ATs on their daily healthcare work, and which roles do ATs play in enhancing their tasks and responsibilities?
3. Which open needs and barriers HCW experience in ATs deployment and usage.

**Table 1:** Related work on AT benefits in healthcare for HCWs

Outcomes	Technologies	Sources
Positive Medical Outcomes^a^	Virtual/ Mixed/ Augmented Reality	[13]
	Medical Devices with interfaces	[27, 28]
	Service, social assistive surgery robots	[29–33]
	AI-driven solutions	[34]
Education and Training^b^	Virtual/ Mixed/ Augmented Reality	[13]
	Social assistive robots	[32]
Enhanced Safety and Quality of Care^c^	Virtual/ Mixed/ Augmented Reality	[13]
	Communication tools and TMed	[9, 28, 35, 36]
	Decision Support Systems	[34, 37]
	Service robots	[29]
	Electronic Health records	[28, 38]
Enhanced Healthcare Access^d^	Communication tools and TMed	[9, 39, 40]
	Electronic health record	[41]
Reduction of HCW Burden and Costs^e^	Communication tools and TMed	[9, 36, 39]
	Decision Support Systems	[42]
	Service and Social robots	[3, 29, 31, 43]
	Electronic health records	[38]
	Sensors and wearables	[40, 44]

^a^ Surgery management, cognitive enhancement, infection protection, drug adherence ^b^ Training, increased patient activity, disease coping, behavioral support ^c^ Reducing human error ^d^ Remote treatment, patient monitoring ^e^ Reduced physical demand, less stress, data exchange, resource allocation, more care time

## Methods

To address the RQs, we conducted an online survey on HCW ATs perceptions during August 2024. The study population incorporated currently employed HCW in Bavaria, Germany, recruited via Bavaria’s health regions. HCWs of different professions, such as administrators, nurses, or doctors, and various areas, such as inpatient and outpatient care, were surveyed. The method integrates quantitative survey analysis with qualitative story completion tasks to better understand HCW experiences.

### Recruitment and inclusion of survey participants

Data for this research was collected cross-sectionally in August 2024. Approval to conduct this human research was granted under file number 2024-06-V-201-R by the joint ethics commission of Bavarian universities (GEHBA). No remuneration or reward was offered for participation in the study. The inclusion criteria focused on HCWs currently employed in Bavaria who provided voluntary and informed consent. The exclusion criteria encompassed individuals not working in healthcare, retired or on leave HCWs, and those unable or unwilling to provide informed consent.

A sample size calculation was initially performed to estimate representativeness for specific HCWs. For example, with over 18,000 general practitioners and specialists in Bavaria [54], a minimum of 377 doctors was needed to achieve a 95 % confidence intervall (CI). While this calculation provided a benchmark, formal representativeness was not the primary goal due to the reliance on convenience sampling. Nurses, an even larger group, require even greater participation rates, which proved challenging. We focused on maximizing the sample size by reaching all accessible HCWs.

We distributed our online survey through the Bavaria’s health regions, a network of health institutions around Bavaria. We used convenience sampling, contacting all available inpatient and outpatient institutions via the health region network. This network-driven recruiting via the health regions ensured that the survey was only provided to HCWs such as nurses, assistant nurses, doctors, or administrators to minimize distortion and to control the study population (see Appendix, Table A1). The survey response rates were monitored during the study period, and we reminded the HCWs on August 15^th^, 2024, to participate.

### Online survey and validation of study items

We collected data through an online survey distributed among HCW, consisting of 14 questions in four sections. Section (i) included demographics (sex, age, knowledge about technologies, work conurbation, professional role, work area), (ii) utilization of ATs (frequency of different soft- and hardware), (iii) perceived usefulness (rank of different soft- and hardware) and (iv) story stem (challenges, solutions). Quantitative areas (i-iii) were extended by a qualitative area (iv) with a story completion task, following the method by Braun et al. [55]. We used two story stems, as shown in Table 2, prompting HCW to reflect on their challenges and potential ATs solutions by finishing a story about how AT could impact their job after attending a trade fair.

**Table 2:** Story stems used in the survey for HCW to explore challenges and solutions

Part	Story Introduction
Stem^a^	As a healthcare employee, you can organize your daily tasks more effectively. Together with your colleagues, you have identified the biggest challenges in your work, which are...
Stem^b^	You recently visited a trade fair where you were presented with a solution to your problems. The technological advances presented opened up new, promising possibilities by...

^a^ Focus on identifying the biggest challenges faced by HCWs. ^b^ Focus on exploring technological solutions

The survey is based on the author’s experience and previous work on AT in healthcare [19, 27, 56]. Categories for different AT based on Mohammadnejad et al. [27], covering digital solutions like EHRs, TMed, robotics, or wearables were included. We differentiate between hard and software to better understand the distinct challenges and usage associated with each. Furthermore, we had close-ended and open-ended questions. We chose the story completion method by Braun et al. [55] to access unconscious attitudes and less socially desirable responses, providing richer insights into HCW perspectives. Survey validation was also tested before the pre-test with five participants (three nurses, one doctor, and one administrator) to assess the clarity and relevance of the questions. Regarding the timing, the survey was filled out on average under eight minutes, depending mainly on the scope of the written stories.

In this subsection, we note that participants submitted their responses anonymously, ensuring confidentiality and encouraging honest feedback. No identifying information was collected. All responses were anonymized after extracting anchor examples from the qualitative data to remove any potential identifiers before analysis.

### Quantitative and qualitative evaluation

This study employs a mixed-method evaluation, combining quantitative and qualitative approaches. Quantitative questionnaire responses were analyzed using SPSS (V29, IBM) for descriptive statistics and crosstabs to identify trends. We compared professional groups (e.g., nurses vs. doctors) to explore variations in AT adoption and perceptions. For the $\chi ^2$-square tests as part of bivariate statistics, we set $\alpha = 0.05$ for significance and $\alpha = 0.01$ for high significance, aiming for a 95 % confidence in minimizing the $\beta$ error.

For the open-ended, qualitative data, we employed a deductive template analysis (Table 3) guided by the frameworks of Mayring [57] and Braun and Clarke [58]. To ensure unbiased analysis, researchers were blinded to participant details, such as the healthcare profession, during the coding process. Multiply, we went through free text using F4 Analysis (Dr. Dresing & Pehl GmbH, V1.4) to explore categories narratively. The analysis of story completion employed story maps and counted categories as recommended by Braun et al. [55]. Super- and subcategories were deductively formed from the text modules in insights answering our RQs. Anchor examples were extracted, meaning representative quotes to summarize the results. The category system consisted finally of five main themes, including (i) technology challenges and solutions in (ii) communication, (iii) coordination, (iv) ATs for direct patient care, (v) ATs for indirect patient care and (vi) technology adoption.

**Table 3:** Categories of the template analysis (Overview of Expressed Challenges and Solutions)

Categories	Subcategories	Description
Technology Challenges	Outdated Equipment	Legacy systems, limited functions, instabilities
	Poor Communication	bad interoperability, double work, information loss
	Low Digital Literacy	Staff struggles with new tech
	Incomplete Digitalization	Security Risks and reliance on paper
Communication	Digital Scheduling	Centralized scheduling
	Telehealth	Remote consultations, digital patient interaction
	Knowledge Sharing	Shared digital resources
	Team Communication	Internal communication tools
	Departmental Interfaces	Cross-departmental communication
	Centralized Platforms	Unified systems for information sharing
Coordination	Digital Workflows	Automate tasks, digitize records
	Task Automation	Routine tasks (e.g., data entry)
	Process Visualization	Visualize task progress
ATs for Direct Patient Care	Equipment Updates	Upgrade hardware/software
	Monitoring	Wearables for health tracking, alerts and alarms
	AI Integration	AI through e.g., speech recognition, fall detections
	Robotics	Assistive robots, lifting aids
	Patient Self-Service	Patients enter data, Patient navigation
	Digital Records	Full electronic documentation
ATs for Indirect Patient Care	Bureaucracy Reduction	Streamline administrative work
	Documentation	Automate records, medication management
	Scheduling	Digital tools for shift and patient scheduling
Technology Adoption	Program Customization	Tailored software
	Data Security	Protect data, ensure privacy
	Training	Improve tech skills
	Resource Allocation	Tools to optimize resources, costly

This study excludes non-technology-related challenges, such as job attractiveness or job demands

## Results

According to the survey structure, we first present the (i) study population, (ii) utilization of AT, and (iii) usefulness of AT. Qualitative results will be added to this, mainly quantitative parts. Section (v) follows with solutions and ATs potentials.

### Study population characteristics

As Table 4 vizualizes, a total of 371 HCW (♀ 63.9 %, ♂ 36.1 %) participated in the study. Most participants (58.6 %) describe their technical skills as advanced and second as beginners (31.9 %). Regarding the conurbation of the workplace, less than 9.8 % of participants work in rural areas of 5,000 residents. The majority (38.4 %) of HCW work in urban areas with more than 100.000 residents. Among the respondent’s job roles, 38,6 % worked in administration. 27 % in nursing and 7.5 % as specialist doctors. The share of other roles was lower than these percentages. The largest group works in hospital administration (24.8 %) and inpatient nursing care (18.7 %).

**Table 4:** General characteristics of study participants (*N*=371)

Variable	Category	n ( %)^a^
Gender	Female	237 (63.9 %)
	Male	134 (36.1 %)
Technical Skills^b^	No skills	7 (1.9 %)
	Beginner	118 (31.9 %)
	Advanced	217 (58.6 %)
	Expert	28 (7.6 %)
Urbanization ^c^	Below 5,000 residents	36 (9.8 %)
	5,001 to 20,000 residents	84 (22.9 %)
	20,001 to 100,000 residents	106 (28.9 %)
	Over 100,000 residents	141 (38.4 %)
Professional Role^d^	Specialist doctor	26 (7.5 %)
	Assistant doctor	8 (2.3 %)
	Nursing professional	93 (27.0 %)
	Assistant nurse	23 (6.7 %)
	Administrator	133 (38.6 %)
	Management	20 (5.8 %)
	Other therapists	26 (7.5 %)
	Education and science	16 (4.6 %)

^a^ Valid percentages used, without missings ^b^ Missing Data = 1 ^c^ Missing Data = 4 ^d^ Missing Data = 26

We also collected metrics on age and work experience. Our HCW are, on average, 37.74 years old (SD = 13.96). The 95 % CI for the mean age ranged from 36.31 to 39.18 years. The distribution of ages was moderately positively skewed (skewness = 0.54, SE = 0.13), which suggests that more HCW were younger.

The average company affiliation was 14.15 years (SD = 13.32), with a median of eight years. The experience ranged from zero to 50 years.

### Utilization of AT in the workplace: software

Table 5 summarizes the usage of various software in HCWs for occupational usage.

**Table 5:** AT software usage (*N*=371, sorted by most daily usage, without missing values)

Software	Daily n (%)	Weekly n ( %)	Monthly n ( %)	Never n ( %)
Communication Platforms^a^	278 (82.2 %)	43 (12.7 %)	12 (3.6 %)	5 (1.5 %)
Text Editors^b^	231 (68.3 %)	69 (20.4 %)	27 (8.0 %)	11 (3.3 %)
Calendar/Scheduling^c^	224 (66.3 %)	57 (16.9 %)	27 (8.0 %)	30 (8.9 %)
Electronic Health Record^d^	174 (52.3 %)	47 (14.1 %)	27 (8.1 %)	85 (25.5 %)
Spreadsheets^e^	136 (40.4 %)	98 (29.1 %)	66 (19.6 %)	37 (11.0 %)
Knowledge Management^f^	129 (38.6 %)	104 (31.1 %)	62 (18.6 %)	39 (11.7 %)
Other Software^g^	114 (34.4 %)	56 (16.9 %)	48 (14.5 %)	113 (34.1 %)
Hospital Information Systems^h^	97 (29.2 %)	49 (14.8 %)	34 (10.2 %)	152 (45.8 %)
Translation ^i^	58 (17.4 %)	91 (27.2 %)	120 (35.9 %)	65 (19.5 %)
Voice-to-Text^j^	36 (10.8 %)	57 (17.2 %)	29 (8.7 %)	210 (63.3 %)
Decision Support Systems^k^	34 (10.2 %)	48 (14.4 %)	38 (11.4 %)	213 (64.0 %)
Patient Education^l^	32 (9.6 %)	76 (22.8 %)	65 (19.5 %)	160 (48.0 %)
Televisits/-medicine/-nursing^m^	29 (8.7 %)	52 (15.7 %)	42 (12.7 %)	209 (63.0 %)

^a^ e.g., WhatsApp, MS Teams, email ^b^ e.g., Word ^c^ e.g., duty rosters ^d^ e.g., care documentation tools ^e^ e.g., Excel ^f^ e.g., UpToDate ^g^ e.g., applications not specified elsewhere, like time or room management software ^h^ e.g., CGM Clinical ^i^ e.g., Google Translate ^j^ e.g., speech recognition software ^k^ e.g., diagnostic suggestions ^l^ e.g., informational applications for patients ^m^ e.g., video conferencing tools for remote consultations

Over 82.8 % HCWs state that they use communication platforms daily. Next to that, 68,3 % text editors, 66.3 % calendars including scheduling software, and 52,3 % electronic health records are used daily. Knowledge software (31.1 %) and spreadsheet programs (29.1 %) are mostly used weekly. Furthermore, translation software (35.9 %) and spreadsheets (19.6 %) are the most common regarding monthly usage. Regarding software never used, HCWs answered in descending order with nearly two-thirds decision support systems, voice-to-text solutions, and remote consultations. HCWs furthermore uses special applications like room planning, lab software, analyzing software for psychological diagnostics, or even AI four times. In addition, we examined whether software usage varies between demographics, such as gender and the professional role. Every software was tested via $\chi ^2$-test for independence with gender. The only category that indicated a significant weak correlation with gender was other software that HCWs added ($\chi ^2$ value = 7.943,*p* = 0.047, Cramer’s V = 0.155).

As the Appendix explains, software use depends on HCWs role. All software, except communication platforms, knowledge management, TMed, translation, and other software, show significance in crosstabulations with roles. Highly significant ( $\alpha < 0.01$) dependence with the job role was examined in communication platforms, text editors, EHRs, hospital information systems (HIS), and patient education software.

Crosstabs, summarized in Table 6 shows that *Admins*, *Nurses* and *Doctors*, which are the most common roles in our study population, depend on the usage of communication platforms. 96.2 % of doctors, 84.9 % of admins and 74.7 % of nurses use communication platforms daily. 80.7 % of *admins* use second scheduling/calendar software and 73.9 % text editors daily. Regarding *nurses*, 72.5 % second use EHRs and 58.2 % scheduling/calendar software. *Doctors*, in comparison to nurses, use text editors (76.9 %) more commonly, e.g., to issue medical letters, and after that EHRs (57.7 %).

**Table 6:** Top 3 daily used software (SW), sorted by job roles, % within job roles

Role	1^st^	n	2^nd^	n	3^rd^	n
Admins	Communic.^b^	101 (84.9%)	Scheduling^a,c^	96 (80.7%)	Text Ed.^d^	88 (73.9%)
Nurses	Communic.^b^	68 (74.7%)	EHR^a^	66 (72.5%)	Scheduling^a,d^	53 (58.2%)
Doctors	Communic.^b^	25 (96.2%)	Text Ed.^d^	20 (76.9%)	EHR	15 (57.7%)

^a^variable not significant in $\chi ^2$-test ^b^ Communication Platforms ^c^ Scheduling/ Calendar ^d^ Text Editors

### Utilization of AT in the workplace: hardware

Table 7 summarizes hardware usage for occupation-related tasks. In alignment with communication software being the most used software, communication hardware, including phones or nursing call systems, has 86.0 %, even higher daily usage. Next to that, 70.7 % HCWs use the printer, daily. 45.1 % daily use sensors in the building, and 44.9 % daily use devices for patient data recording, such as tablets with documentation apps. Weekly, the printer (22.2 %) and the fax (20.5 %), both traditional hardware of decades, were predominantly used. In third place, scanners with text recognition capabilities are used by 19.9 %. HCWs expressed the fax (28.6 %), scanner (12.7 %) and identification devices such as smartcards (10 %) as monthly used devices. Concluding this paragraph with rarely used tech, more than three-quarters never used physical robots (85.6 %). Categories that more than half of the participants never used included other hardware not listed in the table, identification devices, and medical devices with interfaces like a blood sugar measuring that transfers the values to the EHR.

Regarding other additional hardware with 22.8 %, HCWs reported in the open-ended text field, HCWs mostly use standard computers and rarely, in six instances, HCW used specialized hardware, e.g., 3D-Scanners or Exoskeletons.

**Table 7:** AT hardware usage (*N*=371, sorted by most daily usage, without missing values)

Hardware/Devices	Daily n (%)	Weekly n ( %)	Monthly n ( %)	Never n ( %)
Communication ^a^	288 (86.0 %)	24 (7.2 %)	10 (3.0 %)	13 (3.9 %)
Printer^b^	236 (70.7 %)	74 (22.2 %)	21 (6.3 %)	3 (0.9 %)
Sensors in the Building^c^	148 (45.1 %)	41 (12.5 %)	23 (7.0 %)	116 (35.4 %)
Patient Data Recording^d^	149 (44.9 %)	50 (15.1 %)	23 (6.9 %)	110 (33.1 %)
Med. Devices without Interfaces^e^	127 (38.3 %)	49 (14.8 %)	28 (8.4 %)	128 (38.6 %)
Scanners^f^	113 (34.0 %)	66 (19.9 %)	42 (12.7 %)	111 (33.4 %)
Body-Worn Sensors^g^	92 (28.0 %)	40 (12.2 %)	28 (8.5 %)	168 (51.2 %)
Med. Devices with Interfaces^h^	91 (27.7 %)	42 (12.8 %)	17 (5.2 %)	179 (54.4 %)
Fax^i^	87 (26.2 %)	68 (20.5 %)	95 (28.6 %)	82 (24.7 %)
Other Hardware^j^	73 (22.8 %)	28 (8.8 %)	24 (7.5 %)	195 (60.9 %)
Identification Devices^k^	65 (19.6 %)	37 (11.2 %)	33 (10.0 %)	196 (59.2 %)
Physical Robots^l^	8 (2.5 %)	19 (5.8 %)	20 (6.1 %)	279 (85.6 %)

^a^ e.g., mobile phones, tablets ^b^ e.g., office or ward printers ^c^ e.g., motion sensors, smoke detectors ^d^ e.g., tablets used for patient records ^e^ e.g., blood pressure monitors without digital interfaces ^f^ e.g., document scanners with text recognition ^g^ e.g., wearable ECG monitors, smartwatches ^h^ e.g., infusion pumps with digital interfaces ^i^ e.g., traditional fax machines ^j^ e.g., unspecified devices like tablets, laptops ^k^ e.g., fingerprint scanners, e-health cards ^l^ e.g., transport robots, robotic arms for patient assistance

We also examined the dependence of hardware usage on gender and professional roles. Except for other hardware ($\chi ^2$ value = 10.504,*p* = 0.015, Cramer’s V = 0.181), there is no significant effect between hardware and gender. Even the effect size of Cramer’s V indicates a small effect. In conclusion, male and female HCWs use similar ATs, and bivariate statistics are explained in the Appendix for completeness.

For the job role and hardware usage $\chi ^2$-tests showed significant dependence on medical devices with and without interfaces, scanners, body-worn sensors, fax, and identification devices (see Appendix). Crosstabulation is summarized in Table 8. *Admins* (84.9 %), *nurses* (87.9 %) use communication devices, which align with the frequent communication software use. *Doctors* use printers most (92.3 %) and second communication devices (84.6 %). In the second rank *admins* and *nurses* use printers mostly. In the third rank, building sensors (*admins*) and medical devices without interfaces (*nurses* and *doctors*) are reported. In comparison, medical devices with interfaces, e.g., vital measurements connected to the EHR, were reported rare. 38.6 % nurses use devices with interfaces daily, and 61.5 % of doctors.

**Table 8:** Top 3 daily used (HW), sorted by job roles, % within job roles

Role	1^st^	n	2^nd^	n	3^rd^	n
Admins	Communic.^a,b^	98 (82.4%)	Printer^a^	88 (74.6%)	Build. Sensor^a,c^	61 (52.1%)
Nurses	Communic.^a,b,a^	80 (87.9%)	Printer^a^	59 (64.8%)	Med. Devs^d^	45 (50%)
Doctors	Printer^a^	24 (92.3%)	Communic.^a,b,a^	22 (84.6%)	Med. Devs^d^	20 (76.9%)

^a^variables not significant in $\chi ^2$-test ^b^ Mobile Communication Devices ^c^ Sensors in the Building ^d^ Medical Devices without Interfaces

### Usefullness of AT

**Fig. 1:**
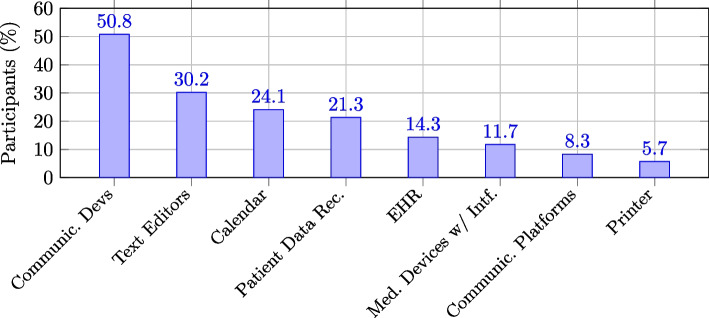
Soft- and hardware ranked as most beneficial for the job (*N*=315, only values > 5 %)

HCWs were asked to rate of various ATs helping HCWs to do a better job, summarized in Fig. 1. 95 times (30.2 %), text editors were mentioned first as the most beneficial software. Moreover, calendar/scheduling programs (24.1 %), EHRs (14.3 %), and communication platforms (8.3 %) are highly seen to be beneficial. Regarding hardware, mobile communication devices were mentioned 160 times (50.8 %) first as the most beneficial hardware. Next, follow top-notch tools to better manage mobile patient data recording (21.3 %) and medical devices with interfaces (11.7 %). In comparison, medical devices without interfaces were ranked at 3.2 % first.

In addition, we examined whether software and hardware usage varies between demographics, such as gender and job role. Crosstabulation in the usefulness of AT shows that both male and female HCWs graded high text editors, calendars/planning programs, and EHRs. $\chi ^2$-test for independence indicated no significant association between gender and self-reported AT usefulness ($\chi ^2$ value = 14.808, *p* = 0.191, Cramer’s V = 0.217), as the assumptions of the chi-square test were not met. Specifically, 11 cells (45.8 %) had an expected count of less than 5, with the minimum expected count being 0.75. This suggests that the data did not fulfill $\chi ^2$ requirements. We also examined the dependence of hardware usage on gender and job roles. A $\chi ^2$ test showed similar software usage, with no significant association between gender and hardware rated as beneficial ($\chi ^2$ value = 8.263, *p* = 0.603, Cramer’s V = 0.162).

Both differences were explored regarding the job role and perceived hard- and software utilization. It depends on the job role and which software is most perceived as beneficial ($\chi ^2$ value = 116.153, *p* = 0.003, Cramer’s V = 0.237). We also found significance and moderate correlation in hardware and job role ($\chi ^2$ value = 99.339, *p* = 0.012, Cramer’s V = 0.219). As Table 9 visualizes, *administrators* and *doctors* see the most utility in text editors. Nurses rate EHRs as most useful. According to Table 10 states that *nurses* and *admins* mainly can do a better job with communication platforms. For *doctors* medical devices with interfaces are seen as most useful. *Nurses* rely more on devices without interfaces showed by their ratings.

**Table 9:** Most beneficial software sorted by job roles, % within job roles

Role	1^st^	n	2^nd^	n	3^rd^	n
Admins	Text Ed.	36 (32.1%)	Scheduling	28 (25.0%)	Communic.^b^	12 (10.7%)
Nurses	EHR	26 (29.9%)	Scheduling	21 (24.1%)	Text Ed.	19 (21.8%)
Doctors	Text Ed.^a^	7 (30.4%)	HIS^b^	5 (21.7%)	Scheduling	3 (13.0%)

^a^ Communication Platforms ^b^ Hospital information systems, e.g. CGM Clinical

**Table 10:** Most beneficial hardware sorted by job roles, % within job roles

Role	1^st^	n	2^nd^	n	3^rd^	n
Admins	Communic.^a^	58 (51.8%)	Pat. Data^b^	25 (22.3%)	Med. Devs.^c^	9 (8.0%)
Nurses	Communic.^a^	35 (40.2%)	Pat. Data^b^	26 (29.9%)	Med. Devs.^d^	6 (6.9%)
Doctors	Med. Devs.^c^	10 (43.5%)	Communic.^a^	7 (30.4%)	Pat. Data^b^	4 (17.4%)

^a^ Mobile Communication Devs. ^b^ Mobile Patient Data Recording ^c^ Medical Devs. with Interfaces ^d^ Medical Devs. without Interfaces

### Story completion: challenges of HCW

A total of 241 entries were made for the first story stem, which asked HCWs to continue a scenario detailed in Table 2, describing challenges they encounter in their work.

#### Communication as main challenge

Forty six (19.09 %) comments voiced communication as the main challenge for HCWs. The ATs interoperability is hindered and depends on manual redundant tasks. Despite EHRs being available for the entire hospital, HCWs often needs to transcribe data manually into EHRs and depend on phone calls to chase data from other departments. Coordination of patient care, e.g., scheduling diagnosis and intersectoral therapy appointments, is challenging (Stem 1, par. 216). Information is often not transferred automatically, leading to faults, wrong prioritization, and a lack of information (Stem 1, par. 196). This also accounts for the challenging knowledge management (Stem 1, par. 180). Missing information distracts and leads to inefficiencies (Stem 1, par. 180). In addition to communication issues between departments and interprofessional communication, the team’s internal communication is expressed as challenging in 14 (5.81 %). This includes communicating holidays, sick leaves, and unmet care needs between the shifts of different HCWs. The following quotes show challenges in...

*’Communication [...] require considerable time and can lead to errors.’* (Admin ^♀^, 21 yr)^1^

*’Missing interfaces (e.g., between lab, patient, diagnostics, patient transport) lead to long waiting times and repeated transports of the patient, which would not be necessary with correct timing and would save resources.’* (Nurse ^♀^, 36 yr)^2^

*’Store, maintain, and constantly renew shared knowledge.’* (Other Therapists ^♀^, 36 yr)^3^

(1) Stem 1, par. 131 — (2) Stem 1, par. 196 — (3) Stem 1, par. 180

### Outdated, insufficient and inconsistent technologies

As a challenge, outdated ATs with limited functionality, such as a missing interface to the EHRs or an app for mobile devices, are mentioned. Furthermore, infrastructure like Wi-Fi, the availability of LAN slots, power sockets, and bandwidth in healthcare facilities are often described as insufficient. Typical hard- and software need to be modernized and improved in usability according to the HCWs. For example, the time-consuming documentation task is done via care documentation software, but other software or paper is also needed to document all parameters. Nurses, doctors, and so on use different software. Because of missing interfaces, information is often exchanged by printing or faxing a doctor’s letter, lab reports, material orders, or, e.g., patient transport documents. When new ATs are introduced, it’s not guaranteed that interfaces to, e.g., EHR exist, leading to inefficiencies (Stem 1, par. 142). In addition, data is transferred manually from devices for vital sign measurement into the documentation software and EHRs. Data security is also challenging when dealing with inconsistent and outdated ATs.

*’Outdated tech that could be modernized to simplify work processes.’* (Admin ^♀^, 29 yr)^1^

*’The problem is that the tech often fails. This is very time-consuming. If the Internet doesn’t work or EHR doesn’t function, it complicates daily tasks.’* (Other ^♀^, 44 yr)^2^

*’The biggest challenge is that the technical aids do not work properly and thus prevent us from focusing fully on the recovery of the patients.’* (Nurse ^♀^, 19 yr)^3^

(1) Stem 1, par. 71 — (2) Stem 1, par. 232 — (3) Stem 1, par. 54

Moreover, the framework ATs used in healthcare has limitations, such as budgets, staff shortages, and digital literacy, which is a reason for outdated solutions.

### Inefficient workflows and documentation

HCWs frequently cite documentation as time-consuming and note that paper and manual processes persist despite digital systems. The lack of optimized and consistent ATs supported workflows is mentioned. Waiting times due to administrative delays occur (Stem 1, par. 216). Documentation is a significant challenge, and HCWs expresses the burden of administrative tasks. Data is mainly entered manually and can be a source of faults. This aligns with expressions that digitalization is not consistently implemented, resulting in media breaks where transitions between paper and digital systems disrupt workflows, causing information loss and inefficiencies. Not using ATs for TMed also leads to inefficiencies due to travel. Due to nonconsistent documentation, examinations are made twice, which wastes capacities (Stem 1, para 216).

*’The large paper load despite the digital documentation [...] contracts are still signed in paper form, then have to be scanned and integrated into the digital file.’* (Nurse ^♀^, 27 yr)^1^

*’There are too many steps involved, such as the 100 clicks for storage.’* (Nurse ^♀^, 49 yr)^2^

(1) Stem 1, par. 186 — (2) Stem 1, par. 239

#### Digital literacy and concerns

Negative perceptions and stigmata exist regarding ATs usage by elderly HCWs and patients (Stem 1, par. 113). ’The elderly have challenges getting used to technology’ (Stem 1, 83) is one of the many sentences that HCWs express. Skepticism towards digital tools is related to missing information, skills, leadership, and education. This leads to a slow improvement of existing ATs and outdated equipment in terms of speed, safety, and capabilities. Nevertheless, although concerns such as data security, privacy, and questions regarding data curation exist, the benefits outweigh most HCWs.

*’Few employees are familiar with the new tech.’* (Admin ^♀^, 33 yr)^1^

*’Older colleagues and bosses are skeptical about newer technologies.’* (Nurse ^♀^, 27 yr)^2^

(1) Stem 1, par. 89 — (2) Stem 1, par. 78

### Story completion: solutions and potential of AT

In addition to identifying challenges, 233 HCWs provided solutions by continuing a hypothetical scenario where they attended an AT trade fair, envisioning the types of solutions they wanted to see. Thereby, ATs are described as beneficial for efficiency, relief to nurses, facilitating state-of-the-art healthcare, and improving HCWs working conditions and job attractiveness (Stem 2, par. 170). Especially integrated systems and automated tools for patient monitoring, documentation, and communication are helpful (Stem 2, par. 108). With ATs, more significant costs through better coordination can be saved while increasing quality and safety (Stem 2, par. 108).

#### ATs for direct patient care

Most of the comments (56.22 %) express to solve the problems with ATs for direct patient care. HCWs voiced concerns about equipment updates, including updated procedures with modern patient monitoring ATs to relieve HCWs from repetitive tasks (Stem 2, par. 88). This can include ATs wearables for fall recognition or the absence of dementia patients (Stem 2, par. 7). Alarm for emergencies should be directly on the HCWs phone or on corridor screens (Stem 2, par. 64, 135). Biometrical identification and QR codes were also helpful (Stem 2, par. 193). Vital signs and other data should be automatically transferred into documentation software and care records to avoid mistakes and wasting time by typing values in manually (Stem 2, par. 15, 133). Therefore, all devices that measure parameters need interfaces (Stem 2, par. 216). Furthermore, robots can also assist in direct patient care, for example, in mobilizing patients and relieving HCWs from physical burden (Stem 2, par. 57).

*’Via scanning of QR codes automatically assign data to patients.’* (Admin ^♀^, 26 yr)^1^

*“[...] detect patients at risk of falling using sensors and accompany them safely from A to B and raise the alarm if the situation requires nursing support”* (Nurse ^♀^, 56 yr)^2^

*’Systems with traffic lights to indicate the urgency would be helpful.’* (Nurse ^♀^, 64 yr)^3^

*’The values are transferred automatically, and different departments [...] allowing patient records from the emergency room to be used on the general ward. Listed items or injuries [...] can be directly integrated into the assessment tools on the general ward (e.g., size, weight, aids like glasses or hearing aids in the electronic patient record).’* (Nurse ^♀^, 24 yr)^4^

*’A robot can mobilize patients weighing over 100 kg more easily.’* (Nurse ^♀^, 31 yr)^5^

(1) Stem 2, par. 116 — (2) Stem 2, par. 231 — (3) Stem 2, par. 215 — (4) Stem 2, par. 193 — (5) Stem 2, par. 57

#### ATs for indirect patient care

Indirect care should also be technically supported (Stem 1, par. 57). Study participants state that the documentation software must be improved through voice commands, warnings, and mobile device possibilities (Stem 2, par. 215). Some HCWs have exact imaginations of how such a system should look like, e.g., as ’mobile portable inductively charging laptops with touch, voice input, camera, spelling correction and adaptive hardware keyboard with software tailored to the therapy process” (Stem 2, par. 178). HCWs state that technical problems must be reduced and servers and end devices must be improved. HCWs prefers user-friendly ’all-for-one’ solutions that include as many usages as possible, such as care documentation, recipes, labs, scanning, and invoicing (Stem 2, par. 157). Generally, participants’ voices for visualization of processes via software, e.g., gant charts and patient pathways (Stem 2, par. 98). Administration, including documentation and billing, can be supported via AI, which helps with automatic text suggestions, spell checking, speech recognition and semantic search functions (Stem 2, par. 173). AI also helps to verify data, automatically translate, and do the billing (Stem 2, par. 55). AI can be used for decision support in diagnostics and triage in the emergency room and to enhance patient monitoring and care prioritization (Stem 2, par. 128, 133). In addition, robots were mentioned for physical assistance in transporting goods to free up time for HCWs (Stem 2, par. 57). Especially the initial admission and small talk with patients as well as the lifting of patients or transporting hospital goods are suggested be carried out or accompanied by robots (Stem 2, par. 133). Medication management is a potential use case for robots, preparing medication blisters automatically (Stem 2, par. 220).

*’It helps when mobile apps support the documentation to save time.’* (Nurse ^♀^, 26 yr)^1^

*’Software that converts speech into text.”* (Nurse ^♀^, 61 yr)^2^

*’A system generates duty rosters using AI and takes into account requests and absences, such as vacation, of the employees.’* (Admin ^♀^, 25 yr)^3^

*’AI analyzes patient data and presents diagnostic suggestions.’* (Nurse ^♂^, 29 yr)^4^

*’An AI robot can assist in the [...] recording symptoms or diagnosis.’* (Admin ^♀^, 25 yr)^5^

(1) Stem 2, par. 130 — (2) Stem 2, par. 216 — (3) Stem 2, par. 99 — (4) Stem 2, par. 82 — (5) Stem 2, par. 133

#### Coordination and communication tools

ATs for coordination and communication, which are lastly one of the biggest challenges, should be technically supported (Stem 1, 57). Centralized platforms and EHRs are frequently mentioned as tools to improve information flow in real-time (Stem 2, par. 12). EHRs should be accessible across different departments for better information access to vital parameters, diagnoses, and treatments (Stem 2, par. 57, 152). Leveraging remote consultation solutions helps to simplify workflows and bring efficiency by avoiding travel and freeing up time for direct patient care (Stem 2, par. 112, 129). Therefore, devices need quick-dial functionalities and should include internal chats to address patient-related issues faster (Stem 2, par. 124). In addition, HCWs mention AI as support for generating duty rosters (Stem 2, par. 61). With this, teams can track who is available and coordinate better when last-minute changes occur.

*’A central platform for managing all patient-related data enables real-time information exchange, reducing misunderstandings and increasing efficiency.’* (Admin ^♀^, 24 yr)^1^

*’Automate administration and optimize patient pathways enables efficient data management, faster communication, and precise scheduling.’* (Admin ^♂^, 36 yr)^2^

*’Patients can Facetime with relatives, and HCWs can communicate.’* (Nurse ^♀^, 46 yr)^3^

(1) Stem 2, par. 49 — (2) Stem 2, par. 74 — (3) Stem 2, par. 121

#### Technology adoption

Adopting ATs requires structured training. HCWs emphasized overcoming skepticism and fears of new tech, particularly among older staff, by offering training (Stem 2, par. 170). HCWs express the importance that ATs are reliable, easy to use, and work without glitches that hinder patient care (Stem 2, par. 128, 164). Data security and privacy and security are noted topics that must be met by ATs (Stem 2, par. 183). In all cases, the ATs are needed for relief, meaning that it helps to reduce bureaucracy and effort in daily indirect care tasks, that HCWs have more time to spend on direct patient care (Stem 2 par. 74, 108). Participants furthermore note the finalization of ATs as crucial (stem 2, par. 170).

*’Proven strategies from all other digital data fields must also be applied to medicine (24-hour IT support, backup server, adequate infrastructure, test operation of updates/bug fixes/patches in a sandbox before a clinic-wide rollout.’* (Doctor ^♂^, 37 yr)^1^

*’Training and education help to build tech skills and reduce fears. [...] Providing technical support, financial budgets for tech and instructions increases acceptance and trust in digital solutions.’* (Nurse ^♂^, 43 yr)^2^

(1) Stem 2, par. 164 — (2) Stem 2, par. 170

## Discussion

Our research provides insights into HCWs usage and perception of ATs via a cross-sectional online survey in Bavaria, Germany, in August 2024.

### Status quo of the utilization of AT

To answer the first RQ about the utilization of ATs, a majority of the of the surveyed HCWs use communication platforms (82.2 %), text editors (68.3 %), scheduling tools (66.3 %) and EHRs (52.3 %) daily. Communication platforms are also the most common for all HCWs roles (Table 6). Some useful ATs, such as voice-to-text solutions, decision support systems, and televisions, are never used by more than half surveyed HCWs even if literature shows potential in these areas [59]. Regarding hardware, communication devices (86 %) and printers (70.7 %) are used most frequently. However, more advanced ATs, such as robots, decision support systems, and telepresence solutions, are rarely used, with less than 10 % of HCWs using them daily. The widespread acceptance of advanced ATs, such as robots, is not yet as developed or widespread as it is for other ATs [3]. This and missing knowledge can be a reason for the low adoption of advanced ATs voicing the need for training, awareness, and research. The reliance on older ATs, such as fax (used daily by 26.2 %), highlights the modernization need. Peek et al. [60] state that advanced tech, e.g., AI-driven ATs, is in early adoption. Comparing the HCWs perspective with patients, also patient-centered ATs remains low used and especially advanced ATs are underutilized [48].

Our template analysis verifies the intense use of communication tools and reveals that challenges persist due to outdated system integration, confirmed in the literature [28]. Manual processes like printing, faxing, and scanning remain prevalent, contributing to inefficiencies and redundancies (Stem 1, par. 216). Participants emphasized the need for updated, fully integrated systems to streamline workflows and reduce manual tasks, suggesting an improved AT adoption.

### Usefullness and solutions through AT

The second RQ addresses the impact and usefulness of ATs. Different ATs, mobile communication devices, text editors, calendar/scheduling software, and patient data recording were ranked first as beneficial (Fig. 1). Advanced AT were ranked by less than 5 % as most beneficial, probably because HCWs still use ATs like robots rarely. Robots can be helpful but are not yet considered by HCWs [3, 43]. However, our data show that frequently used ATs, particularly those supporting communication, are positively perceived, reinforcing the link between familiarity and perceived usefulness.

Our qualitative findings indicate that ATs contribute to improvements in working conditions, job attractiveness, and care quality (Stem 2, par. 108), aligning prior research on ATs’s enhancement of healthcare documentation, coordination, and communication [60]. Our participants voice the importance of central platforms for communication. Patient monitoring systems, such as vital parameter tracking and fall detection, are crucial for direct patient care. ATs should be integrated into electronic documentation and include mobile applications to deliver their full potential to streamline processes and support HCWs. While individual ATs have been proven to work and relief nurses via TMeds service robotics, our HCWs voice the need for a broader seamless ATs integration across different processes and workflows [3, 19, 35, 43, 61]. HCWs mention solutions like voice-assisted documentation, billing software, decision support systems, automated medication, and intelligent transport robots as examples to maximize ATs outcome for healthcare delivery. An excellent example of an integrated ATs is a patient monitoring system that detects dangerous situations, gives automatic alarms, recommends, and documents via interfaces in the EHRs.

### Challenges of HCW in AT usage and adoption

To answer the third RQ, the main challenges for HCWs are communication and outdated technologies, according to the story stems. Both are confirmed in the literature [60]. Information is lacking between different team members and departments. Also, the tech is missing interfaces and is often outdated. Although quantitatively 58.6 % rate their skills as advanced, in the story stems also occur concerns and facilitation of digital literacy is a core necessity in ATs adoption. Digital literacy, in this context, refers to the ability to use effectively, navigate through, and adopt ATs. Training on ATs is considered essential for tech adoption in our study population. Previous studies showed digital literacy as a primary facilitator for ATs adoption [35, 46]. More than half of our surveyed HCWs stated advanced tech skills, a positive and essential development.

Additional factors are important for ATs, including the need for system integrations and person-centered approaches [62]. In open-ended data, reliability and data safety are cited as important. ATs needs to work flawlessly that HCWs not get frustrated [35, 46]. Participants also stated that ATs needs to provide usability and data safety (Stem 2, par. 170). Furthermore, according to Sommer et al. [35, 43], HCWs should experience positive ATs outcomes, such as work relief, to facilitate adoption.

### Methodological and result limitations

Our sample comprises 371 HCWs, limiting statistical power. Recruitment of HCWs was conducted via the Health Regions Plus, limiting the population to this network of healthcare institutions in Bavaria. The survey utilized a convenience sampling approach, which limits generalizability and precludes the calculation of a precise response rate. The response rate is likely relatively low, as dissemination depended on intermediary institutions’ willingness and capacity to forward the survey to HCWs. Because of the limited study participants, case number requirements for $\chi ^2$ tests were often unmet, leading to limitations in the bivariate exploration of differences between urbanity and ATs usage. Our population also shows that 58,6 % HCWs claimed advanced technical skills, which could be influential, even though we didn’t find a correlation between tech skills and adopting ATs. Digital skills are vital to ATs usage, but infrastructure and system integration are important [63]. We relied on self-reported data, leading to a potential BIAS. Nevertheless, combining close-ended questions with story stems increased the level of detail in our results [64]. Despite limitations, our somewhat indicative than conclusive study reveals the usage, potential, and perception in ATs.

### Recommendations and further research

Despite the study’s limitations, such as the sample size, we suggest that research effort needs to go into care processes and workflow design to seamlessly integrate novel ATs, improving communication, information flow, and overall patient care by allowing HCW to spend more time with patients. All workflows need to be created with reliability and usability in mind. The new processes need fault controllability and account for complete disruption to avoid improvisation that can put patients at risk. The success of ATs adoption will be tight to the digital literacy of HCWs. Therefore, the usability of fully integrated ATs will facilitate more accessible learning and overall better adoption. Further research is required to explore long-term outcomes, trust-building mechanisms, and the role of organizational support in ATs adoption. We also recommend further advanced ATs development and implementation to maximize their potential for improving healthcare delivery. The underutilization stands in contrast to previous work on nurses, which showed that advanced ATs in the form of AI is seen by two-thirds as an opportunity [19].

## Conclusion

With our survey-based study, in summary, we explored the usage and perception of ATs among HCWs. The findings highlight the widespread adoption and positive perception of basic ATs, such as communication devices and information management tools, while revealing the underutilization of advanced ATs, such as robotics and AI.

Communication remains a challenge for HCWs, despite the high availability of ATs for information flow. Qualitative insights pointed to interoperability failures, manual redundant tasks, and poor coordination across departments, contributing to inefficiencies. Although tools like patient data recording devices and medical equipment are widely used, outdated tech like fax machines (used daily by 75.3 % of HCWs) or printers (used daily by 99.1 % HCWs) persist, underscoring a need for further digitalization. Basic ATs are well-integrated, and advanced ATs, such as body-worn sensors and robots, are used by less than one-third, with physical robots being the least adopted ATs, concluding further work in this area.

Our research qualitatively also revealed the potential benefits of ATs in relieving HCWs workloads and improving efficiency. However, familiarity and experience with ATs are hampered by outdated infrastructure and insufficient integration, leading to inefficiencies and additional manual work. To address these challenges, improving the usability, reliability, and integration of ATs into healthcare workflows is critical. HCWs indicated that ATs need to empower them to focus on patient care. The lack of training time and the fear of technical failures also emphasize the need for easy-to-learn, robust, and reliable ATs that minimize the adoption barriers. ATs have the potential to improve healthcare, but further research with larger samples is needed better to understand their adoption and operation within healthcare workflows to maximize their positive impact.

## Supplementary Information

Supplementary Material 1. We append the survey and additional data. The data sets generated for the study are available on request.

## Data Availability

We append the survey and additional data. The data sets generated for the study are available on request.

## Materials availability

The survey is available in the Appendix and can be used when citing the authors.
