# Supplementary material for: Assistive technologies in healthcare: utilization and healthcare workers perceptions in Germany
Source: BMC Health Serv Res. 2025 Feb 10;25:223. doi: 10.1186/s12913-024-12162-x (PMC11812205; doi:10.1186/s12913-024-12162-x)
Supplement: Supplementary file 1 — Supplementary Material 1. We append the survey and additional data. The data sets generated for the study are available on request. [file 12913_2024_12162_MOESM1_ESM.pdf]

Appendix A Inclusion Criteria for the study

Table A1 visualizes the inclusion- and exclusion.

Table A1 Inclusion and Exclusion Criteria

Inclusion Criteria	Exclusion Criteria
HCW working in healthcare in Bavaria.	Individuals not working in healthcare
Currently working and employed HCW.	Former, retired, or on sick leave HCW
Voluntarily and informed consent	Informed consent not given or unable

Appendix B Questionnaire

The following appendix lists the questions of our online survey on assistive technologies in healthcare to provide a comprehensive overview and offer a tool for other authors in related research.

B.1 Introduction

Study on Technologies in Healthcare - *An initiative by the Deggendorf Institute of Technology, FAU Erlangen-Nuremberg, and Augsburg Institute of Technology.*

Welcome! Your opinion on assistive technologies is essential to us!

Key Information on the Survey:

- All healthcare professionals from all regions of Germany are warmly invited.
- The goal is to obtain a nationwide perspective on technologies in healthcare.
- The survey takes approximately 10 minutes to complete.
- Your responses are collected in a privacy-friendly manner and under high-security standards.

Thank you for your support!

B.2 Demographic Data

1. What is your gender?

   ☐ Male ☐ Female ☐ Diverse

2. What is your age?

   ----

3. How would you rate your knowledge of new technologies?
   ☐ No skills
   ☐ Beginner
   ☐ Advanced
   ☐ Expert

4. In which conurbation do you work?
   ☐ Under 5,000 inhabitants
   ☐ 5,001 to 20,000 inhabitants
   ☐ 20,001 to 100,000 inhabitants
   ☐ Over 100,000 inhabitants
5. How long have you worked in healthcare?
   ____
6. What is your role in care?
   ☐ Specialist doctor
   ☐ Assistant doctor
   ☐ Nursing professional
   ☐ Nursing assistant
   ☐ Administration (medical specialists, documentalists, etc.)
   ☐ Other: ____________________
7. Your area of work can be categorized as follows:
   ☐ Inpatient nursing care (e.g., clinics/rehab)
   ☐ Doctor in a clinical environment (e.g., hospital doctor)
   ☐ General practitioner
   ☐ Emergency room
   ☐ Intensive care
   ☐ Administration/management in the hospital environment
   ☐ Administration/Management in elderly care
   ☐ Other: ____________________

B.3 Utilisation of Digital Solutions

1. What digital solutions do you use in your professional environment? (Software)
   - Text editors (e.g., Word)
     ☐ Daily
     ☐ Weekly
     ☐ Monthly
     ☐ Never
   - Spreadsheets (e.g., Excel)
     ☐ Daily
     ☐ Weekly
     ☐ Monthly
     ☐ Never
   - Calendar/Scheduling (e.g., duty rosters)
     ☐ Daily
     ☐ Weekly
     ☐ Monthly
     ☐ Never
   - Communication platforms (e.g., WhatsApp, MS Teams, e-mail, chatbots)
     ☐ Daily
     ☐ Weekly
     ☐ Monthly

☐ Never

- Patient education software
  - ☐ Daily
  - ☐ Weekly
  - ☐ Monthly
  - ☐ Never
- Electronic health record/care documentation
  - ☐ Daily
  - ☐ Weekly
  - ☐ Monthly
  - ☐ Never
- Hospital information systems (HIS/ERP such as CGM Clinical)
  - ☐ Daily
  - ☐ Weekly
  - ☐ Monthly
  - ☐ Never
- Voice-to-text programs
  - ☐ Daily
  - ☐ Weekly
  - ☐ Monthly
  - ☐ Never
- Software for telemedicine
  - ☐ Daily
  - ☐ Weekly
  - ☐ Monthly
  - ☐ Never
- Decision support systems (e.g., diagnostic suggestions)
  - ☐ Daily
  - ☐ Weekly
  - ☐ Monthly
  - ☐ Never
- Knowledge management (e.g., UpToDate)
  - ☐ Daily
  - ☐ Weekly
  - ☐ Monthly
  - ☐ Never
- Translation software
  - ☐ Daily
  - ☐ Weekly
  - ☐ Monthly
  - ☐ Never
- Other: ____________________
  - ☐ Daily
  - ☐ Weekly
  - ☐ Monthly
  - ☐ Never

2. What digital solutions do you use in your professional environment? (Hardware)
   - Mobile communication (e.g., mobile phones, DECT)
     - ☐ Daily
     - ☐ Weekly
     - ☐ Monthly
     - ☐ Never
   - Mobile patient data recording (e.g., tablets)
     - ☐ Daily
     - ☐ Weekly
     - ☐ Monthly
     - ☐ Never
   - Medical devices without interfaces
     - ☐ Daily
     - ☐ Weekly
     - ☐ Monthly
     - ☐ Never
   - Medical devices with interfaces
     - ☐ Daily
     - ☐ Weekly
     - ☐ Monthly
     - ☐ Never
   - Body-worn sensors
     - ☐ Daily
     - ☐ Weekly
     - ☐ Monthly
     - ☐ Never
   - Sensors in the building
     - ☐ Daily
     - ☐ Weekly
     - ☐ Monthly
     - ☐ Never
   - Fax
     - ☐ Daily
     - ☐ Weekly
     - ☐ Monthly
     - ☐ Never
   - Printer
     - ☐ Daily
     - ☐ Weekly
     - ☐ Monthly
     - ☐ Never
   - Scanner with OCR
     - ☐ Daily
     - ☐ Weekly
     - ☐ Monthly
     - ☐ Never

- Physical robots
  - ☐ Daily
  - ☐ Weekly
  - ☐ Monthly
  - ☐ Never
- Identification systems
  - ☐ Daily
  - ☐ Weekly
  - ☐ Monthly
  - ☐ Never
- Other: ____________________
  - ☐ Daily
  - ☐ Weekly
  - ☐ Monthly
  - ☐ Never

B.4 Perceived Usefulness

1. Rank the software that helps you do your job better. (Select up to 3)
   - ☐ Text editors
   - ☐ Spreadsheets
   - ☐ Calendar/Scheduling
   - ☐ Communication platforms
   - ☐ Patient education software
   - ☐ Electronic health records
   - ☐ Hospital information systems
   - ☐ Voice-to-text programs
   - ☐ Telemedicine
   - ☐ Decision support systems
   - ☐ Knowledge management
   - ☐ Translation software
2. Rank the hardware that helps you do your job better. (Select up to 3)
   - ☐ Mobile communication
   - ☐ Mobile patient data recording
   - ☐ Medical devices without interfaces
   - ☐ Medical devices with interfaces
   - ☐ Body-worn sensors
   - ☐ Sensors in the building
   - ☐ Fax
   - ☐ Printer
   - ☐ Scanner with OCR
   - ☐ Physical robots
   - ☐ Identification systems

B.5 Story Stems

Please continue writing the story. There are no limits. Be yourself. For example, challenges in daily work can be described in the first part of the story. The second part can then show solutions. (Please dont write names and other personal data in the fields)

1. As a healthcare employee, you have the freedom to organize your daily tasks more effectively. Together with your colleagues, you have identified the biggest challenges in your work, which are primarily...

   ----
2. Fortunately, you recently visited a trade fair where you were presented with a solution to your problems. The technological advances presented opened up new, promising possibilities by ...

B.6 Thank you!

You have contributed significantly to research into assistive technologies in healthcare. With your input, we can develop solutions for current challenges.

Appendix C Bivariate Statistics

Table C2 Bivariate Statistics between Software and Gender

**Software**	χ^2 **Value**	**Cramer's V**
Text editors	χ^2 value = 1.749, p = 0.626	Cramer's V = 0.072
Spreadsheets	χ^2 value = 2.511, p = 0.473	Cramer's V = 0.086
Calendar/Scheduling	χ^2 value = 0.624, p = 0.891	Cramer's V = 0.043
Communication Platforms	χ^2 value = 3.689, p = 0.297	Cramer's V = 0.104
Patient education software	χ^2 value = 4.839, p = 0.184	Cramer's V = 0.121
Electronic Health Record	χ^2 value = 5.352, p = 0.148	Cramer's V = 0.127
Hospital Information Systems	χ^2 value = 3.919, p = 0.270	Cramer's V = 0.109
Voice-to-text programs	χ^2 value = 3.113, p = 0.375	Cramer's V = 0.097
Televisits-/medicine/nursing	χ^2 value = 0.703, p = 0.873	Cramer's V = 0.046
Decision support systems	χ^2 value = 4.848, p = 0.183	Cramer's V = 0.121
Knowledge management	χ^2 value = 0.711, p = 0.871	Cramer's V = 0.046
Translation programmes	χ^2 value = 5.204, p = 0.157	Cramer's V = 0.125
Other software*	χ^2 value = 7.943, p = 0.047	Cramer's V = 0.155

* Includes software not categorized elsewhere; only significant value, $\alpha = 0.05$ for significance

Table C3 Bivariate Statistics between Hardware and Gender

Hardware/Devices	χ^2 Value	Cramer's V
Mobile Communication	χ^2 value = 7.464, p = 0.058	Cramer's V = 0.149
Patient Data Recording	χ^2 value = 2.361, p = 0.501	Cramer's V = 0.084
Med. Devices without Interfaces	χ^2 value = 0.505, p = 0.918	Cramer's V = 0.918
Med. Devices with Interfaces	χ^2 value = 2.066, p = 0.559	Cramer's V = 0.079
Body-Worn Sensors	χ^2 value = 2.748, p = 0.432	Cramer's V = 0.092
Sensors in the Building	χ^2 value = 0.994, p = 0.803	Cramer's V = 0.055
Fax	χ^2 value = 0.750, p = 0.861	Cramer's V = 0.048
Printer	χ^2 value = 2.750, p = 0.432	Cramer's V = 0.091
Scanners	χ^2 value = 2.825, p = 0.419	Cramer's V = 0.092
Physical Robots	χ^2 value = 5.160, p = 0.160	Cramer's V = 0.126
Identification Devices	χ^2 value = 5.973, p = 0.113	Cramer's V = 0.134
Other Hardware*	χ^2 value = 10.504, p = 0.015	Cramer's V = 0.181

* Includes hardware not categorized elsewhere; only significant value, $\alpha = 0.05$ for significance

Table C4 Daily Usage of Software by HCW roles (N=371, sorted by total usage)

Daily Software Usage	Admins n (%)	Nurses n (%)	Doctors n (%)	Total
Communication Platforms[-]	101 (38.7 %)	68 (26.1 %)	25 (9.6 %)	261
Text editors[1]	88 (40.9 %)	51 (23.7 %)	20 (9.3 %)	215
Calendar/Scheduling[2]	96 (45.5 %)	53 (25.1 %)	13 (6.2 %)	211
Electronic Health Record[3]	52 (30.2 %)	66 (38.4 %)	15 (8.7 %)	172
Spreadsheets[4]	68 (54.4 %)	25 (20.0 %)	6 (4.8 %)	125
Knowledge management[-]	49 (40.5 %)	32 (26.4 %)	14 (11.6 %)	121
Other software[-]	40 (38.5 %)	28 (26.9 %)	12 (11.5 %)	104
Hospital Information Systems[5]	36 (38.3 %)	34 (36.2 %)	10 (10.6 %)	94
Translation programmes[-]	29 (54.7 %)	8 (15.1 %)	3 (5.7 %)	53
Patient education software[6]	14 (45.2 %)	6 (19.4 %)	5 (16.1 %)	31
Voice-to-text programs[7]	15 (42.9 %)	5 (14.3 %)	6 (17.1 %)	35
Decision support systems[8]	16 (47.1 %)	12 (35.3 %)	1 (2.9 %)	34
Televisits-/medicine/nursing[-]	12 (42.9 %)	6 (21.4 %)	5 (17.9 %)	28

[-] No significance in χ^2-tests % within each software category [1] χ^2 value = 53.107, p = 0.000, Cramer's V = 0.237 [2] χ^2 value = 48.256, p = 0.001, Cramer's V = 0.226 [3] χ^2 value = 55.823, p = 0.000, Cramer's V = 0.245 [4] χ^2 value = 77.605, p = 0.000, Cramer's V = 0.287 [5] χ^2 value = 32.656, p = 0.050, Cramer's V = 0.187 [6] χ^2 value = 33.994, p = 0.036, Cramer's V = 0.191 [7] χ^2 value = 50.220, p = 0.000, Cramer's V = 0.232 [8] χ^2 value = 34.065, p = 0.036, Cramer's V = 0.191

Table C5 Daily Usage of Hardware by HCW Roles (N=371, sorted by total usage)

**Daily Hardware Usage**	**Admins n (%)**	**Nurses n (%)**	**Doctors n (%)**	**Total**
Mobile Communication[-]	98 (36.3 %)	80 (29.6 %)	22 (8.1 %)	270
Printer[-]	88 (39.3 %)	59 (26.3 %)	24 (10.7 %)	224
Patient Data Recording[-]	49 (34.5 %)	41 (28.9 %)	14 (9.9 %)	142
Sensors in the Building[-]	61 (43.3 %)	37 (26.2 %)	8 (5.7 %)	141
Med. Devices without Interfaces[1]	26 (21.5 %)	45 (37.2 %)	20 (16.5 %)	121
Scanners[2]	53 (50.0 %)	22 (20.8 %)	8 (7.5 %)	106
Body-Worn Sensors[3]	41 (46.6 %)	23 (26.1 %)	3 (3.4 %)	88
Med. Devices with Interfaces[4]	20 (23.0 %)	34 (39.1 %)	16 (18.4 %)	87
Fax[5]	24 (28.9 %)	24 (28.9 %)	15 (18.1 %)	83
Other Hardware[-]	21 (33.9 %)	16 (25.8 %)	11 (17.7 %)	62
Identification Devices[6]	25 (40.3 %)	8 (12.9 %)	16 (25.8 %)	62
Physical Robots[-]	2 (28.6 %)	3 (42.9 %)	0 (0.0 %)	7

[-] No significance in χ^2-tests [1] χ^2 value = 64.853, $p = 0.000$, Cramer's V = 0.264 [2] χ^2 value = 32.780, $p = 0.049$, Cramer's V = 0.187 [3] χ^2 value = 43.171, $p = 0.003$, Cramer's V = 0.217 [4] χ^2 value = 58.745, $p = 0.000$, Cramer's V = 0.252 [5] χ^2 value = 41.150, $p = 0.005$, Cramer's V = 0.210 [6] χ^2 value = 58.244, $p = 0.000$, Cramer's V = 0.250
